# Comprehensive analysis to identify noncoding RNAs mediated upregulation of maternal embryonic leucine zipper kinase (MELK) correlated with poor prognosis in hepatocellular carcinoma

**DOI:** 10.18632/aging.204059

**Published:** 2022-05-04

**Authors:** ZiYi Guo, Zhitu Zhu

**Affiliations:** 1 Department of Radiology, The First Affiliated Hospital of JinZhou Medical University, Jinzhou, China; 2 Department of Clinical Trial, Institute of Clinical Bioinformatics, Cancer Center of Jinzhou Medical University, The First Affiliated Hospital of Jinzhou Medical University, Jinzhou, China

**Keywords:** noncoding RNA, hepatic cell cancer, MELK, prognosis

## Abstract

Object: Maternal embryonic leucine zipper kinase (MELK) is involved in the development and progression of various cancers. This work investigated the usefulness of MELK in the prediction of hepatocellular carcinoma (HCC) prognosis.

Methods: Information on MELK expression was obtained by pan-cancer analysis using The Cancer Genome Atlas (TCGA) database. The TCGA-liver hepatic cancer (TCGA-LIHC), Oncomine datasets, International Cancer Genome Consortium (ICGC) datasets were used to investigate MELK expression in HCC. The prognostic roles of MELK in HCC were assessed by univariate and multivariate survival analyses. The underlying mechanism for noncoding RNAs (ncRNAs) involved in MELK expression was investigated by *in silico* studies, correlation, methylation, and survival analyses. The relationships between MELK expression and immune cells, immune markers, and checkpoint markers were also analyzed.

Results: (1) MELK was identified as an independent predictor of overall survival (OS) in HCC patients (MELK high vs. low expression, HR 2.469; 95% CI 1.217–5.008; *p* = 0.012) in a multivariate Cox analysis, with a concordance index (C-index) value of 0.727 (95% CI 0.750–0.704). (2) The noncoding RNA miR3142HG and the LINC00265/has-miR-101-3p axis were found to regulate MELK expression in HCC tissue. (3) MELK levels were linked to various immune functions, including tumor infiltration and the expression of immune checkpoints and biomarkers in HCC.

Conclusion: MELK may have an oncogenic function in HCC and was found to be up-regulated by ncRNAs and associated with immune cell infiltration and unfavorable prognosis.

## INTRODUCTION

Hepatocellular carcinoma (HCC) is a common malignancy, particularly in East Asia, and is associated with high mortality [1, 2]. The cancer has a high rate of recurrence, estimated to be between 77% and 100%, largely from the remnant liver, resulting in poor outcomes [3]. The incidence of HCC appears to be increasing, with risk factors including obesity, alcohol abuse, diabetes, chronic viral infection (hepatitis B and C viruses), and metabolic disease [4]. Thus, the determination of biomarkers and targets that will facilitate treatment is necessary.

Maternal embryonic leucine zipper kinase (MELK) is an AMPK serine/threonine kinase [5] and has been proposed as a potential therapeutic target in several cancers [6]. Tang et al. showed that MELK was necessary for proliferation, metastasis, and apoptosis in lung cancer [7], and its use as a diagnostic marker in colorectal [8], ovarian [9], and head and neck cancer has also been reported [10]. MELK promotes mTOR signaling in endometrial cancer [11], and its abnormal expression has been linked to unfavorable breast cancer prognosis [12]. MELK has been found to be targeted by the microRNA (miRNA) miRNA-214-3p in HCC to block proliferation [13]. However, there is as yet no detailed investigation of the role of MELK in HCC, nor is its relationship with immune infiltration of tumors known.

Here, we evaluated the relationship between MELK levels and patient outcomes in a variety of cancers. We also examined MELK regulation in HCC by noncoding RNAs (ncRNAs), as well as an investigation of its function in terms of methylation, tumor infiltration, biomarker expression, and immune checkpoint analysis. The results indicated that up-regulation of MELK by ncRNAs is linked to both tumor infiltration and poor outcome in HCC.

## MATERIALS AND METHODS

### Expression and survival analysis of MELK in the pan-cancer dataset

Data on MELK mRNA expression in 18 cancers were obtained from TCGA (https://genome-cancer.ucsc.edu/). The data were normalized and the package “limma” in R was used to analyze differential expression [13]. A *p*-value < 0.05 was taken as statistically significant. The GEPIA database (http://gepia.cancer-pku.cn/) [14] was used to assess MELK and lncRNA levels in various cancers, again using *p* < 0.05 to represent significance.

### Prediction of MELK-binding miRNAs

We used PITA, RNA22, miRmap, microT, miRanda, PicTar, and TargetScan to predict the potential binding of miRNAs. Candidate miRNAs predicted by two or more programs were used for further analysis. StarBase (http://starbase.sysu.edu.cn/), a database for analyzing miRNAs and their interactions, [15] was used to investigate relationships between miRNA-MELK, lncRNA- MIR3142HG, or lncRNA- LINC00265 in HCC. The levels of hsa-miR-101-3p in tumor and control tissue were determined, as were potential lncRNA candidates for binding to has-miR-101-3p.

### MELK expression in HCC

Gene expression patterns and clinical data pertaining to HCC patients were downloaded from the TCGA database (https://portal.gdc.cancer.gov). RNA-seq data from 375 HCC patients were obtained. MELK expression patterns in HCC were also investigated in the Oncomine dataset and International Cancer Genome Consortium LIRI-JP cohort (*n* = 229) (https://dcc.icgc.org/projects/LIRI-JP). The Human Protein Atlas (HPA, http://www.proteinatlas.org/) was searched for information on MELK protein expression, and information on mutations was obtained from the cBioPortal for Cancer Genomics (http://www.cbioportal.org/). The institutional ethics committee of the First Affiliated Hospital of Jinzhou Medical University approved this study.

### Survival analyses and prognostic model development

Kaplan-Meier curves and log-rank tests were used to measure survival and to assess the prognostic relevance of MELK. Univariate Cox regression was conducted to explore the associations between MELK expression and OS to identify prognostic biomarkers. Multivariate Cox regression was subsequently used to identify factors that were independently associated with outcomes.

### DNA methylation analyses

DNA methylation is controlled by DNA methyltransferases and influences cancer cell behavior. We investigated the expression of DNA methyltransferases in relation to MELK expression in data from the TCGA database. The UALCAN (http://ualcan.path.uab.edu/) and DiseaseMeth v 2.0 (http://bio-bigdata.hrbmu.edu.cn/diseasemeth/) databases were then used to examine MELK expression in HCC tumors and paracancerous tissues and MEXPRESS (https://mexpress.be) [16] was used to determine relationships between MELK and DNA methylation.

### Immune cell infiltration in relation to MELK levels

TIMER (https://cistrome.shinyapps.io/timer/) [17] was used to investigate tumor immune infiltration, specifically, to determine whether there was an association between MELK expression and the infiltration by different types of immune cells. Correlations between MELK levels and the expression of immune checkpoint genes, specifically, CD274, CTLA4, HAVCR2, PDCD1, PDCD1LG2, TIGIT, LAG3, and SIGLEC15, were explored. In addition, the relationships between these variables and MELK copy numbers were assessed in HCC patients, as was their prognostic relevance. Moreover, the correlations between MELK expression and markers associated with 16 different tumor-infiltrating lymphocytes (TILs) were assessed; these included B cells, monocytes, T cells, CD8+ T cells, neutrophils, M1/M2 macrophages, natural killer (NK) cells, DCs, exhausted T cells, Tregs, tumor-associated macrophages (TAMs), and Th1, Th2, Th17, and Tfh cells. Outcome modules were used to investigate relationships between TILs, genomic alterations, and clinical outcomes in the TCGA-LIHC dataset.

### Statistical analysis

Clinicopathological variables associated with MELK expression were analyzed using Pearson chi-squared tests and Fisher’s exact test, as appropriate. Disease-free survival (DFS) was defined as the length of time between surgery and disease recurrence, whereas OS was defined as the length of time from diagnosis to death or the most recent follow-up. Patients for whom these data were not available were excluded from the analysis. Survival outcomes were compared via Kaplan-Meier curves with the log-rank test. Hazard ratios (HRs) with 95% confidence intervals (CIs) were calculated for DFS and OS using univariate Cox proportional hazards regression analysis, with variables found to be significant in the univariate analysis (*p* < 0.05) being incorporated into a multivariate analysis. A two-sided *p* < 0.05 was used as the significance threshold. Analyses were conducted in R (v 3.6) and GraphPad Prism 8.3.

## RESULTS

### MELK expression in pan-cancer

**Figure 1:**
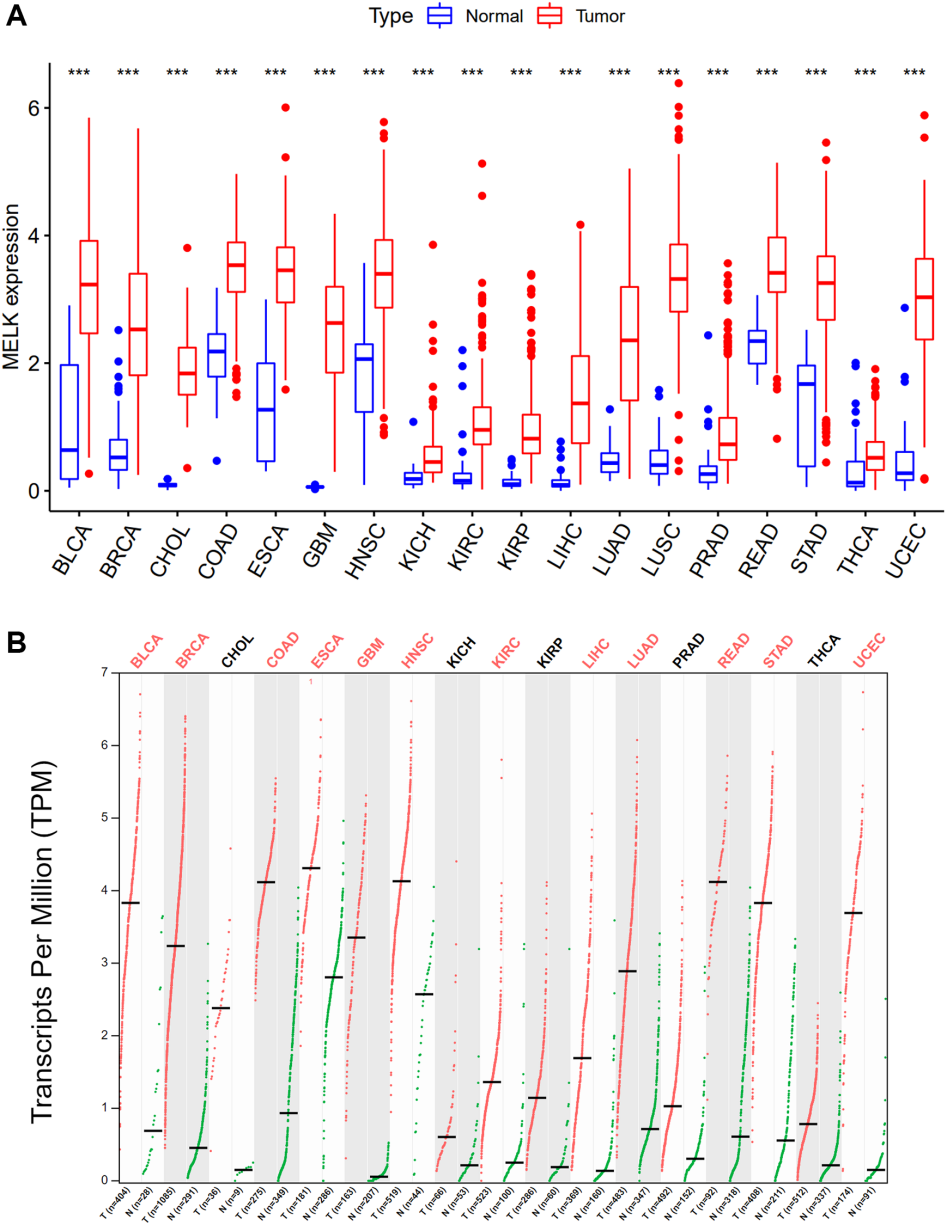
**Expression analysis for MELK in multiple cancers.** (**A**) The expression of MELK in 18 types of human cancer based on TCGA cancer and normal data. (**B**) The expression of MELK in 18 types of human cancer based on TCGA cancer and with corresponding TCGA and GTEx normal tissues. ^*^p value < 0.05; ^**^p value < 0.01; ^***^p value < 0.001.

We initially examined MELK expression in 18 different cancer types, finding that MELK levels were significantly elevated in all 18 cancers in comparison with normal tissue. The cancers investigated included bladder carcinoma (BLCA), breast invasive carcinoma (BRCA), cholangiocarcinoma (CHOL), colon adenocarcinoma (COAD), esophageal carcinoma (ESCA), glioblastoma multiforme (GBM), head-neck squamous cell carcinoma (HNSC), kidney chromophobe (KICH) and kidney renal clear cell carcinoma (KIRC), kidney renal papillary cell carcinoma (KIRP), liver hepatocellular carcinoma (LIHC), lung adenocarcinoma (LUAD), lung squamous cell carcinoma (LUSC), prostate adenocarcinoma (PRAD), rectum adenocarcinoma (READ), stomach adenocarcinoma (STAD), thyroid cancer (THCA), and uterine carcinosarcoma (UCEC). The findings were verified in the GEPIA database, which confirmed the significantly elevated MELK levels (Figure 1). Taken together, MELK was upregulated in all the above cancers, suggesting that it may act as a carcinogenic modulator.

### MELK and pan-cancer prognosis

**Figure 2:**
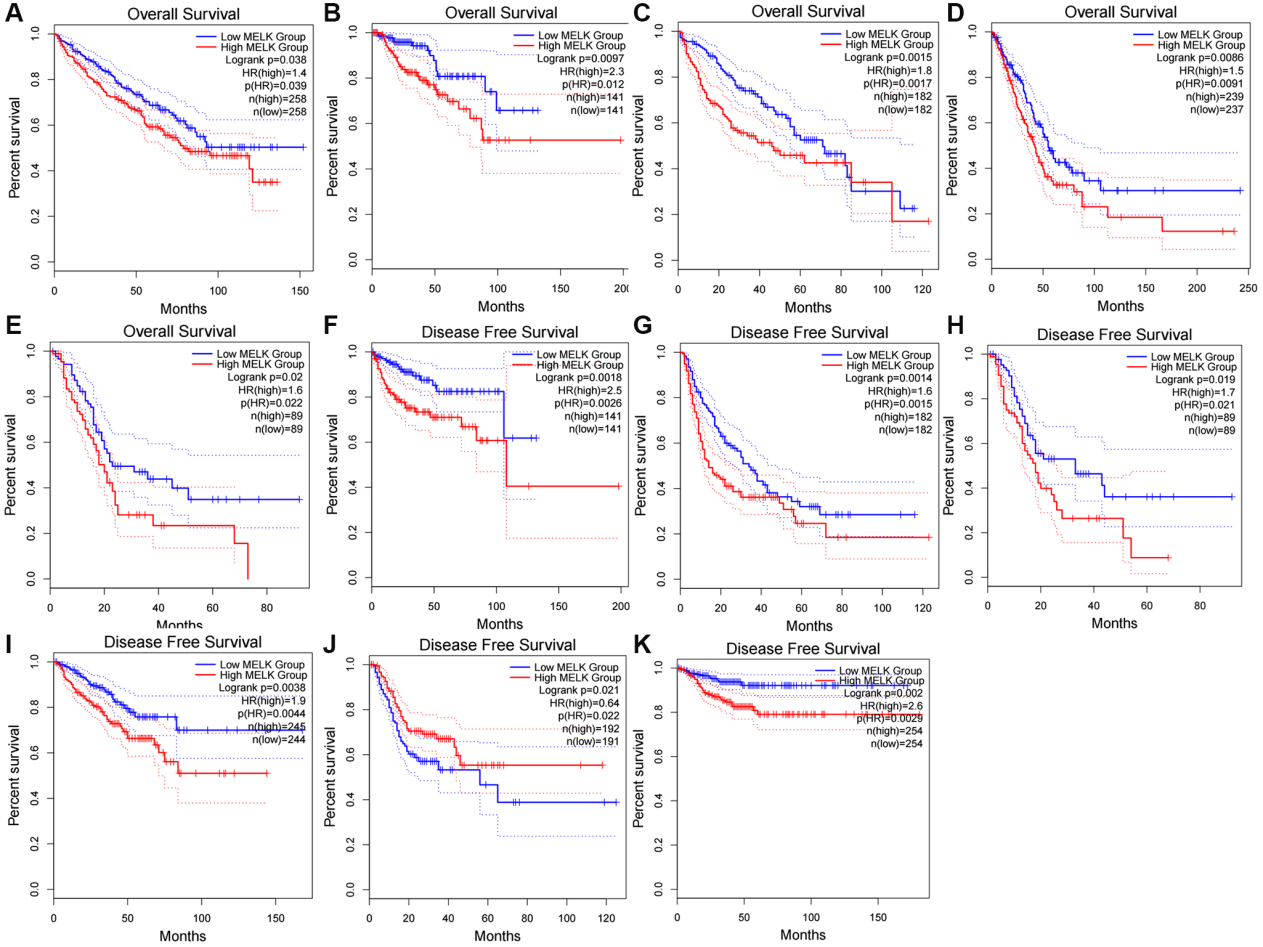
The overall survival (OS) analysis for MELK in various human cancers determined by the “GEPIA” database (**A**–**K**) The OS plot of MELK in KIRC (**A**), KIRP (**B**), LIHC (**C**), LUAD (**D**), and PAAD (**E**); The DFS plot of MELK in KIRP (**F**), LIHC (**G**), PAAD (**H**), PRAD (**I**), STAD (**J**), and THCA (**K**).

The relationship between MELK and survival in the 18 cancer types was evaluated using the GEPIA database, using both overall survival (OS) and disease-free survival (DFS). Elevated levels of MELK in KIRC, KIRP, LIHC, LUAD, and PAAD were linked to poor OS (Figure 2A–2E) while for DFS, the raised expression of MELK in KIRP, LIHC, PAAD, PRAD, STAD, and THCA was found to be associated with poor prognosis (Figure 2F–2K). There were no significant associations between MELK levels and prognosis in the other cancer types. The results suggest the potential of MELK as a biomarker for unfavorable outcomes in HCC.

### Overexpression of MELK in HCC

**Figure 3:**
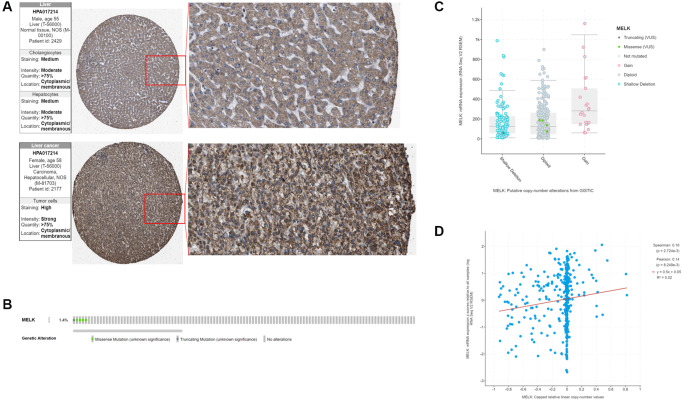
MELK in HCC (**A**) Verification of MELK protein levels using immunohistochemical data from the Human Protein Atlas database. (**B**) MELK genomic alterations in TCGA HCC shown on a cBioPortal OncoPrint plot. Association between MELK copy number and mRNA level shown by dot plot (**C**) and correlation plot (**D**) using cBioPortal.

Having established the abnormal overexpression of MELK in HCC, we then investigated its clinical significance. Kaplan-Meier survival curves (Figure 2D and 2G) showed a significant correlation between MELK overexpression and reduced OS. Data from the Human Protein Atlas (HPA) database indicated overexpression of MELK in HCC tissues compared with normal liver tissue (Figure 3A). In addition, we examined MELK genomic expression and copy number to examine the possible reason for its overexpression. Using cBioPortal, we observed MELK amplification in approximately 1.4% of all HCC samples (Figure 3B), while there was no relationship between the copy number and mRNA levels (Figure 3C and 3D). These results indicate that copy number amplification is not the major mechanism responsible for MELK overexpression in HCC.

### Overexpression of MELK indicated poor prognosis in HCC

We next examined the relationships between MELK and various clinical parameters in the TCGA-LIHC cohort. The basic characteristics of the patients are shown in Table 1. This showed that MELK levels were significantly linked to both tumor diameter (*p* = 0.029) and tumor-node-metastasis (TNM) stage (*p* = 0.032). However, no relationship was observed between MELK and age, sex, BMI, and metastases (both lymph node and distant) (*p* > 0.05; Table 1).

**Table 1:** The expression of MELK and clinicopathologic features in the TCGA-LIHC cohort.

**Characteristic**	**Low expression of MELK**	**High expression of MELK**	***p***
*n*	187	187	
T stage, *n* (%)			< 0.001
T1	110 (29.6%)	73 (19.7%)	
T2	38 (10.2%)	57 (15.4%)	
T3	30 (8.1%)	50 (13.5%)	
T4	6 (1.6%)	7 (1.9%)	
N stage, *n* (%)			0.624
N0	121 (46.9%)	133 (51.6%)	
N1	1 (0.4%)	3 (1.2%)	
M stage, *n* (%)			0.358
M0	129 (47.4%)	139 (51.1%)	
M1	3 (1.1%)	1 (0.4%)	
Pathologic stage, *n* (%)			< 0.001
Stage I	103 (29.4%)	70 (20%)	
Stage II	36 (10.3%)	51 (14.6%)	
Stage III	30 (8.6%)	55 (15.7%)	
Stage IV	4 (1.1%)	1 (0.3%)	
Tumor status, *n* (%)			0.029
Tumor free	112 (31.5%)	90 (25.4%)	
With tumor	66 (18.6%)	87 (24.5%)	
Gender, *n* (%)			0.077
Female	52 (13.9%)	69 (18.4%)	
Male	135 (36.1%)	118 (31.6%)	
Age, *n* (%)			0.088
≤60	80 (21.4%)	97 (26%)	
>60	107 (28.7%)	89 (23.9%)	
BMI, *n* (%)			0.774
≤25	88 (26.1%)	89 (26.4%)	
>25	83 (24.6%)	77 (22.8%)	
Residual tumor, *n* (%)			0.901
R0	168 (48.7%)	159 (46.1%)	
R1	8 (2.3%)	9 (2.6%)	
R2	1 (0.3%)	0 (0%)	
Histologic grade, *n* (%)			< 0.001
G1	39 (10.6%)	16 (4.3%)	
G2	102 (27.6%)	76 (20.6%)	
G3	40 (10.8%)	84 (22.8%)	
G4	4 (1.1%)	8 (2.2%)	
Adjacent hepatic tissue inflammation, *n* (%)			0.496
None	69 (29.1%)	49 (20.7%)	
Mild	51 (21.5%)	50 (21.1%)	
Severe	10 (4.2%)	8 (3.4%)	
AFP (ng/ml), *n* (%)			< 0.001
≤400	127 (45.4%)	88 (31.4%)	
>400	19 (6.8%)	46 (16.4%)	
Albumin (g/dl), *n* (%)			0.834
<3.5	36 (12%)	33 (11%)	
≥3.5	126 (42%)	105 (35%)	
Prothrombin time, *n* (%)			0.069
≤4	103 (34.7%)	105 (35.4%)	
>4	55 (18.5%)	34 (11.4%)	
Child-Pugh grade, *n* (%)			1
A	120 (49.8%)	99 (41.1%)	
B	12 (5%)	9 (3.7%)	
C	1 (0.4%)	0 (0%)	
Fibrosis ishak score, *n* (%)			0.329
0	45 (20.9%)	30 (14%)	
2-Jan	14 (6.5%)	17 (7.9%)	
4-Mar	12 (5.6%)	16 (7.4%)	
6-May	44 (20.5%)	37 (17.2%)	
Vascular invasion, *n* (%)			0.491
No	112 (35.2%)	96 (30.2%)	
Yes	54 (17%)	56 (17.6%)	
OS event, *n* (%)			0.023
Alive	133 (35.6%)	111 (29.7%)	
Dead	54 (14.4%)	76 (20.3%)	
DSS event, *n* (%)			0.042
Alive	152 (41.5%)	135 (36.9%)	
Dead	31 (8.5%)	48 (13.1%)	
PFI event, *n* (%)			0.098
Alive	104 (27.8%)	87 (23.3%)	
Dead	83 (22.2%)	100 (26.7%)	
Age, median (IQR)	62 (52.5, 69)	59.5 (51, 68)	0.136

The median OS for patients with high MELK expression (48.95 ± 8.56 months, 95% CI 32.17–65.73) was significantly shorter than those with low expression (80.68 ± 11.86, 95% CI (57.43–103.93), log-rank *p* < 0.001). Of the 291 cases, 158 patients (54.3%) experienced tumor recurrence during the follow-up period (median time 21 months.55 ± 2.68, 95% CI 16.29–26.81). Recurrence was greater in the group with high MELK expression (106/187) than in the low-expression group (77/187). The median DFS in the high-expression group (13 months 07 ± 2.22, 95% CI 8.71–17.43) was significantly shorter than in the low-expression group (33 months 90 ± 5.13, 95% CI (23.83–43.97), log-rank *p* < 0.001).

#### Analysis of overall survival and MELK expression

Univariate Cox regression showed that OS was associated with MELK expression (MELK high vs. low, HR = 2.308; 95% CI 1.029,5.167; *p* < 0.001), age (> 60 vs. ≤ 60, HR = 2.629; 95% CI 1.398,4.943, *p* = 0.003), vascular invasion (vascular invasion-positive vs. negative, HR = 1.939; 95% CI 0.992,3.789, *p* = 0.053), and tumor status (tumor-free vs. with tumor, HR 2.992; 95% CI 1.557–5.749, *p* = 0.001). All patients showed an association between OS and MELK, age, vascular invasion, and tumor status.

In the multivariate Cox analysis, OS was found to be associated with MELK (MELK high vs. Low, HR 2.469; 95% CI 1.217–5.008, *p* = 0.012), age (age ≤ 60 vs. age > 60, HR 2.568; 95% CI 1.404–4.695; *p* = 0.002), vascular invasion (vascular invasion-positive vs. negative, HR = 2.031; 95% CI 1.067, 3.866, *p* = 0.031), tumor status (tumor-free vs. with tumor, HR 3.267; 95% CI 1.725–6.187, *p* < 0.001). All patients displayed an association of OS with MELK, age, vascular invasion, and tumor status (with tumor or tumor-free), indicating that these were independent prognostic factors for OS. The results are shown in Table 2. The C-index was 0.727 (95% CI 0.75–0.704).

**Table 2:** Univariate and multivariate Cox proportional hazard analyses of MELK expression and overall survival for patients in TCGA-LIHC cohort.

**Characters**	**Univariate analysis**		**Multivariate analysis**	
	**HR (95% CI)**	***p***	**HR (95% CI)**	***p***
Sex (female/male)	0.914 (0.445,1.879)	0.808		
Age (≤65/>65)	2.629 (1.398,4.943)	**0.003**	2.568 (1.404,4.695)	**0.002**
T stage (T1–2 VS. T3–4)	1.467 (0.709,3.034)	0.302		
Vascular invasion	1.939 (0.992,3.789)	**0.053**	2.031 (1.067,3.866)	**0.031**
Tumor Status (tumor free VS. with tumor)	2.992 (1.557,5.749)	**0.001**	3.267 (1.725,6.187)	**<0.001**
AFP	1.058 (0.468,2.393)	0.892		
MELK	2.308 (1.029,5.167)	**0.042**	2.469 (1.217,5.008)	**0.012**

Abbreviations: CI: confidence interval; HR: hazard ratio; AFP: alpha-fetoprotein.

#### Survival analysis for DFS and MELK

Univariate analysis showed associations between DFS and MELK (MELK high vs. low, HR = 3.013; 95% CI 1.592–5.772; *p* < 0.001), tumor stage (T1 and T2 vs. T3 and T4, HR 2.319; 95% CI 1.277–4.212; *p* = 0.006), and tumor status (tumor-free vs. with tumor, HR 6.747; 95% CI 4.091–2.393; *p* < 0.001). These results are listed in Table 2.

In the multivariate analysis, DFS was associated with MELK (HR 2.251; 95% CI 1.274–3.977; *p* = 0.005), tumor stage (T1 and T2 vs. T3 and T4, HR 2.416; 95% CI 1.398–4.175; *p* = 0.002), tumor status (tumor-free vs. with tumor, HR 6.558; 95% CI 4.006–10.738; *p* < 0.001), and AFP (AFP ≤ 200 VS. AFP > 2 00, HR 1.942; 95% CI 1.065–3.541; *p* = 0.030), indicating that these are independent prognostic factors for DFS (all *p* < 0.05; Table 3). The C-index was 0.719 (95% CI 0.693–0.751).

**Table 3:** Univariate and multivariate Cox proportional hazard analysis of MELK expression and disease-free survival (DFS) for patients in TCGA-LIHC cohort.

**Characters**	**Univariate analysis**		**Multivariate analysis**	
	**HR (95% CI)**	***p***	**HR (95% CI)**	***p***
Sex (female/male)	1.402 (0.769,2.467)	0.242		
Age (≤60/>60)	1.447 (0.866,2.416)	0.158		
T stage (T1–2 VS. T3–4)	2.319 (1.277,4.212)	**0.006**	2.416 (1.398,4.175)	**0.002**
Vascular invasion	1.489 (0.884,2.509)	0.135		
Tumor Status (tumor free VS. with tumor)	6.747 (4.091,11.126)	**<0.001**	6.558 (4.006,10.738)	**<0.001**
AFP	1.058 (0.468,2.393)	0.892	1.942 (1.065,3.541)	**0.030**
MELK	3.031 (1.592,5.772)	**<0.001**	2.251 (1.274,3.977)	**0.005**

Abbreviations: CI: confidence interval; HR: hazard ratio; AFP: alpha-fetoprotein.

#### Further evaluation of MELK expression in HCC using the Oncomine and ICGC datasets

**Figure 4:**
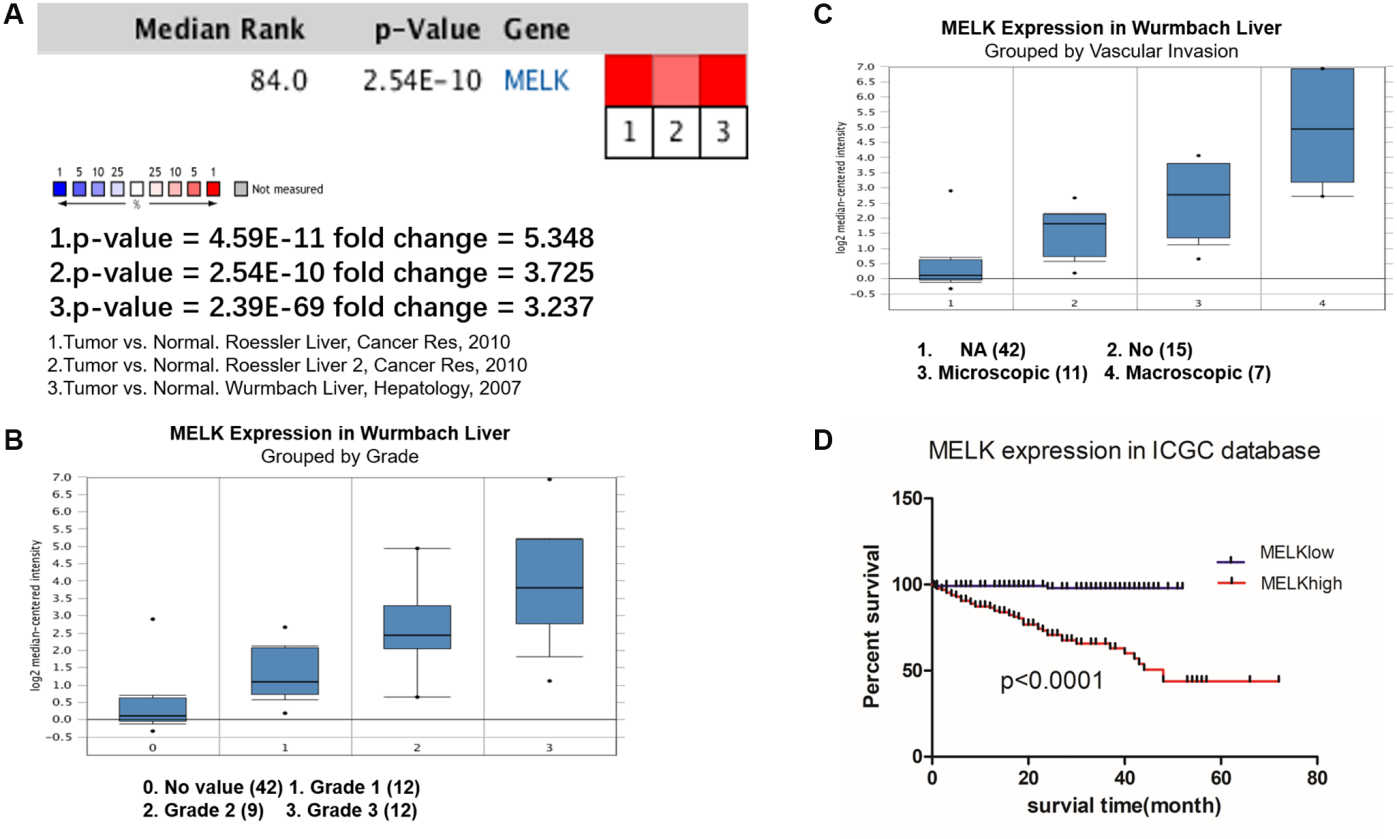
**Oncomine and ICGC database analysis of MELK in HCC.** Oncomine analysis of MELK expression in cancer and normal tissues. (**A**) Heatmaps showing MELK expression in clinical HCC samples vs. normal tissues. Association between MELK expression and tumor grade (**B**) and vascular invasion (**C**) in the Wurmbach Liver dataset. (**D**) Overall survival analysis of MELK expression in the ICGC database.

To further verify the expression pattern and prognostic value of MELK in HCC, we performed meta-analysis using the Oncomine database and survival analysis ICGC database. Oncomine analysis of cancer vs. normal tissue showed that MELK was significantly overexpressed in HCC tissue in different datasets (Figure 4A). In the Wurmbach Liver dataset, higher MELK mRNA levels were associated with both tumor grade and vascular invasion (Figure 4B and 4C). In the ICGC liver cancer dataset, higher expression of MELK was associated with poor OS (Figure 4D).

### miRNAs and MELK expression

**Figure 5:**
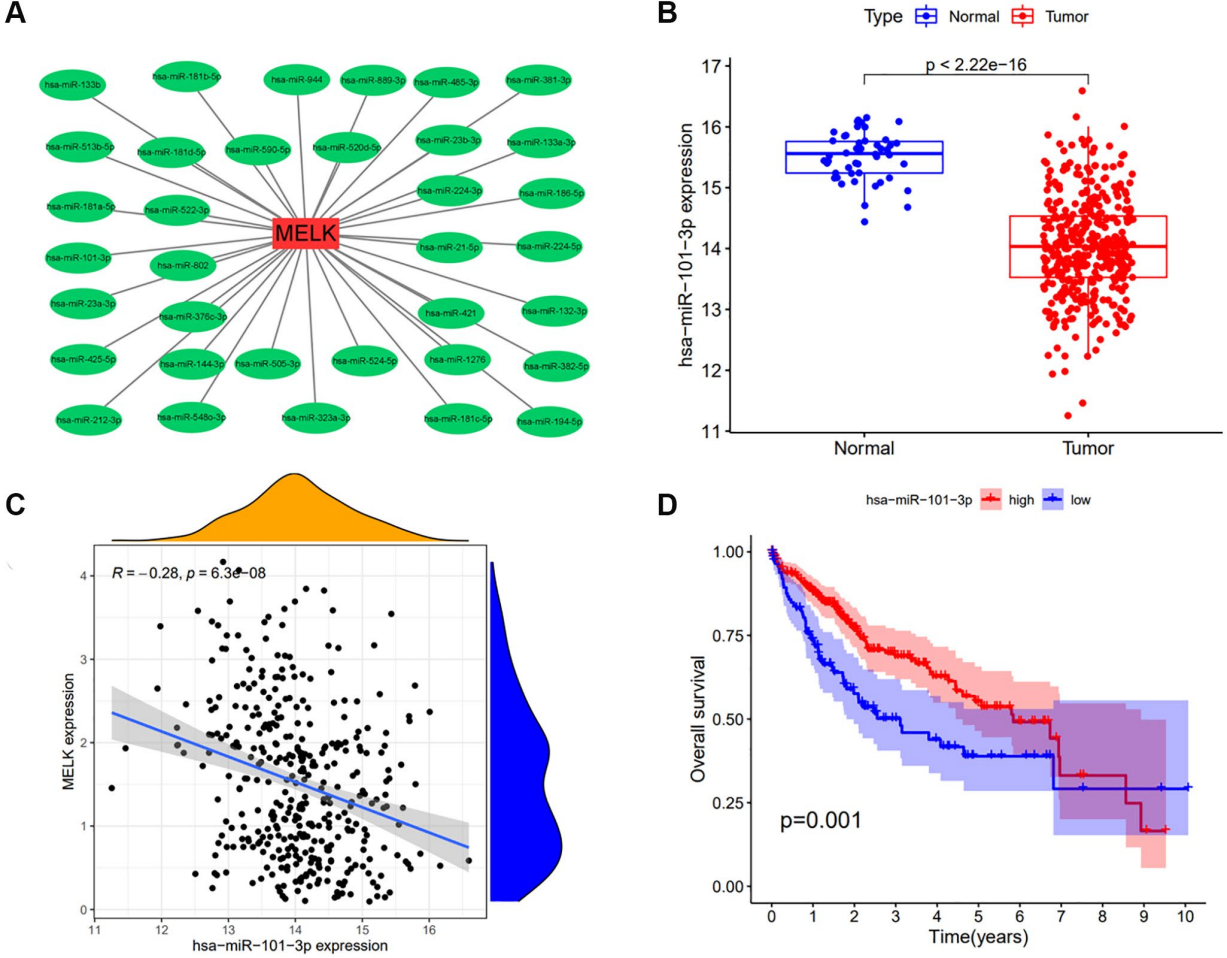
**Analysis of has-miR-101-3p as an upstream regulator of MELK in HCC.** (**A**) miRNA-MELK regulatory network constructed by Cytoscape. (**B**) Association of MELK and miRNA expression analyzed by starBase. (**C**) Expression of hsa-miR-101-3p in tumor and normal tissue analyzed by starBase. (**D**) Prognostic value of hsa-miR-101-3p assessed by Kaplan-Meier survival analysis.

It is well documented that ncRNAs modulate gene expression. To evaluate the possibility that ncRNAs regulate MELK expression, we predicted ncRNAs that could bind to MELK. This yielded 35 miRNAs; Figure 5A shows a visualization of their interactions with MELK determined by Cytoscape (Figure 5A). According to the established mechanism of miRNA-gene interactions, a negative relationship should exist between the miRNA and the MELK level. Investigation of this issue showed a significant negative association between MELK and has-miR-101-3p (Figure 5B and 5C) with a correlation coefficient R = −0.28 (*p* = 6.3e-08), but not between MELK and the remaining 34 miRNAs. We then examined the levels and prognostic ability of hsa-miR-101-3p, observing that miR-101-3p expression was reduced in HCC and that higher levels were associated with better prognosis (Figure 5D). This indicates that miR-101-3p negatively regulates MELK in HCC.

### lncRNAs upstream of has-miR-101-3p

**Figure 6:**
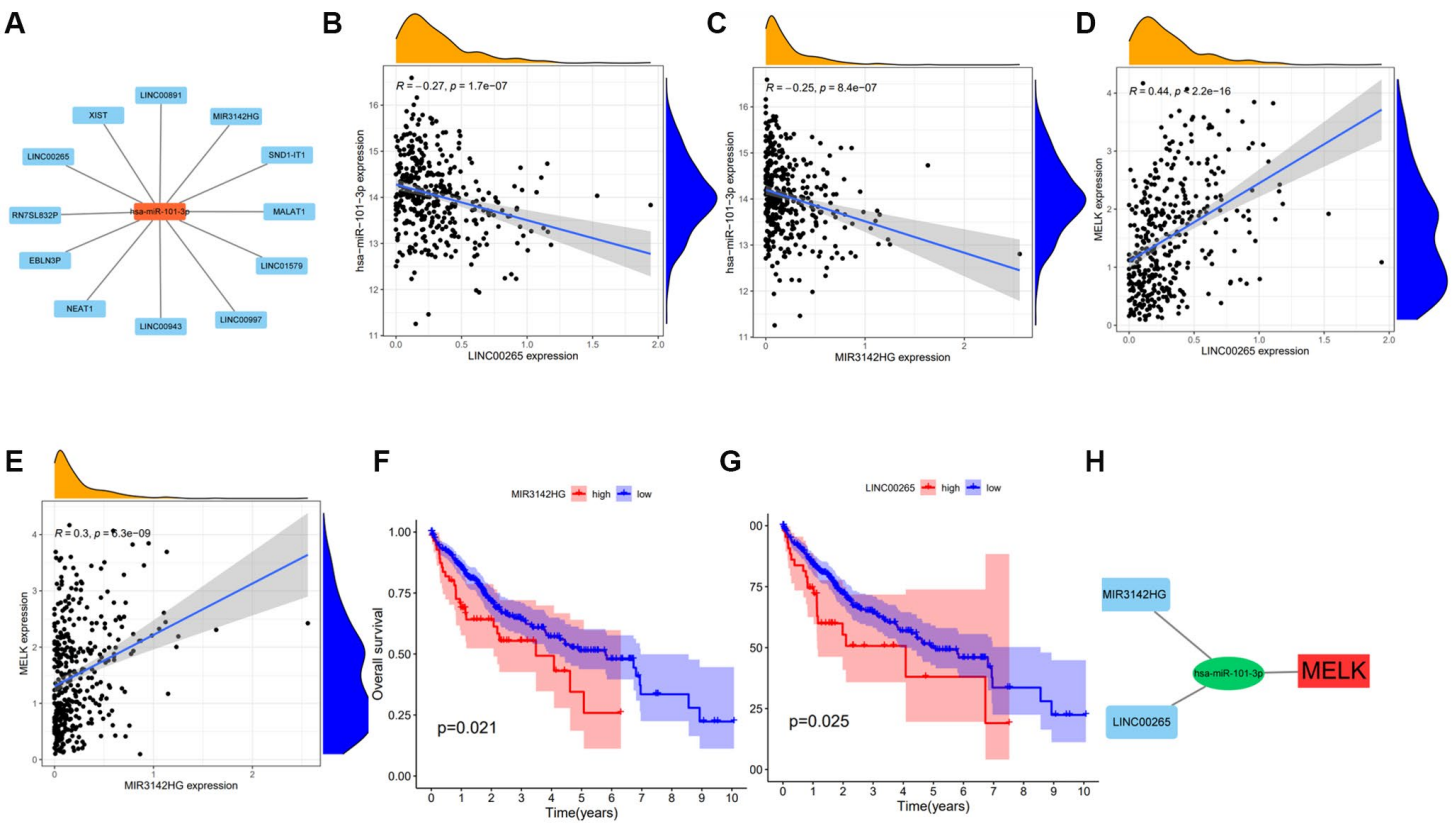
**Expression and survival analysis of upstream lncRNAs of hsa-miR-101-3p in HCC.** 12 types of lncRNAs were significantly associated with hsa-miR-101-3p (**A**). Significant negative correlations were obtained between the levels of LINC00265 (**B**), MIR3142HG (**C**), and has-miR-101-3p. Significant positive correlations were obtained between the levels of LINC00265 (**D**) and MIR3142HG (**E**) and MELK. Overall survival analysis for MIR3142HG (**F**), LINC00265 (**G**) in HCC. (**H**) The MIR3142HG and LINC00265/hsa-miR-101-3p/MELK axis. ^*^p value < 0.05.

We next used StarBase to identify lncRNAs upstream of has-miR-101-3p, and a regulatory network of the 25 predicted lncRNAs and has-miR-101-3p was created using Cytoscape (Figure 6A). Measurement of the levels of these lncRNAs in HCC tissue showed significant negative regulation of only LINC00265 and MIR3142HG (Figure 6B and 6C) while LINC00265 and MIR3142HG positively regulated MELK (Figure 6D and 6E). Raised expression of MIR3142HG and LINC00265 was also found to be linked with reduced OS (Figure 6F and 6G). The competing endogenous RNA (ceRNA) hypothesis proposes that lncRNAs modulate mRNA levels by competitive binding to shared miRNAs. This suggests either a negative relationship between lncRNAs and miRNAs or a positive relationship between lncRNAs and mRNAs. These findings indicate that MIR3142HG and LINC00265 are likely candidates for lncRNAs operating upstream of the hsa-miR-101-3p/MELK axis, as shown in Figure 6H.

### Association between DNA methylation and MELK expression

**Figure 7:**
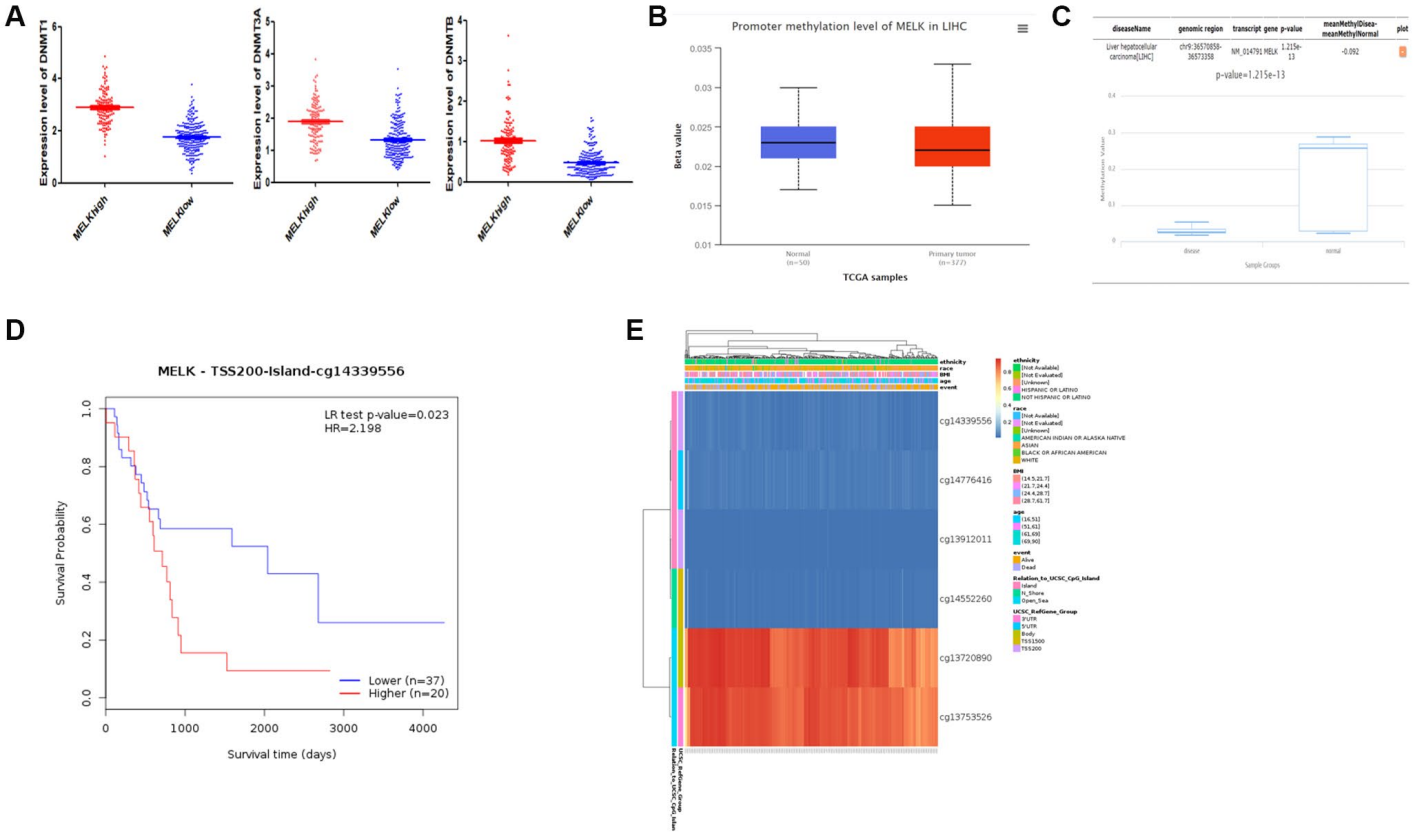
MELK methylation (**A**) Differential expression of three DNA methyltransferases (DNMT1, DNMT3A, and DNMT3B) in the MELK high- and low-expression groups. (**B**) Evaluation of methylation by UALCAN and (**C**) DiseaseMeth version 2.0. (**D**) Survival analysis of methylation sites (cg14339556) in the MELK DNA sequence. (**E**) Analysis of methylation sites visualized by MEXPRESS. The central blue line indicates MELK expression.

To further investigate the mechanisms controlling MELK expression in HCC, we assessed the relationships between MELK levels and methylation. Firstly, we began by comparing the expression of the DNMT1, DNMT3A, and DNMT3B methyltransferases in relation to MELK tumor expression, revealing that all three were up-regulated in the context of higher MELK expression (Figure 7A). Secondly, a UALCAN analysis further revealed a possible link between DNMT1 and increased methylation in normal liver tissue relative to HCC tissue (*p* = 0.101, Figure 7B), while DiseaseMeth v 2.0 analysis showed that MELK methylation was significantly reduced in HCC tumors compared with paired normal tissues (*p* < 0.001; Figure 7C). Hypermethylated regions were present within the 3′- and 5′-UTR regions, whereas the TSS1500 and TSS200 regions tended to be hypomethylated. Thirdly, key methylation sites (eg.cg14339556) in the MELK DNA sequences were negatively associated with MELK expression and poor OS (HR = 2.198, *p* = 0.023, Figure 7D). The differential patterns of MELK expression are represented using heatmaps (Figure 7E).

### Immune cell infiltration and MELK

**Figure 8:**
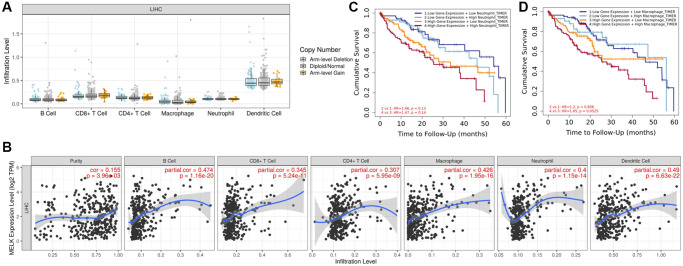
**MELK levels and immune cell infiltration in HCC.** (**A**) Immune cell infiltration in relation to MELK copy number variations. (**B**) Correlation of MELK levels with B cells, CD8+ T cells, CD4+ T cells, macrophages, neutrophils, and dendritic cells. (**C**) Association between MELK levels (HR = 4.636, 95% CI 1.170, 18.369, *p* = 0.029) patient OS at 60 months with increased neutrophil. (**D**) Association between MELK levels and patient OS at 60 months (HR = 6.202, 95% CI 1.578, 24.378, *p* = 0.009) with increased macrophage.

Tumor-infiltrating lymphocytes (TILs) are generally considered to be key independent predictors for lymph node metastasis as well as survival outcomes in many cancer types [17]. Accordingly, we utilized the TIMER database to determine potential links between MELK levels and TILs in HCC. An initial ‘SCNA’ module analysis revealed several infiltrating immune cell populations that were not associated with changes in MELK gene copy number in HCC, including CD4+ T cells, dendritic cells (DCs), B cells, and macrophages (Figure 8A). A subsequent ‘Gene’ module analysis revealed no correlations between MELK and tumor purity, although it was positively linked to B cell, CD8+T, CD4+ T cell, neutrophil, macrophage, and dendritic cell infiltration in HCC (Figure 8B). When we examined the association between MELK levels and patient OS at 60 months, we found that MELK and increased neutrophil (HR = 4.636, 95% CI 1.170, 18.369, *p* = 0.029) and macrophage (HR = 6.202, 95% CI 1.578, 24.378, *p* = 0.009) levels were linked with poorer HCC patient survival outcomes, as shown in Figure 8C and 8D. These findings suggest that MELK may impact HCC patient prognosis and clinical outcomes in part by modulating intra-tumoral immune cell infiltration.

To confirm the relationship between MELK expression and TILs, we examined the levels of immunological marker genes associated with six cell types. This revealed that five of these marker genes, namely, CD19, IRF5, ITGAM, NRP1, and ITGAX which are associated with B cells, M1 macrophages, neutrophils, and dendritic cells, respectively, correlated with MELK levels (Table 4). As such, the interplay between MELK and these immune cell populations may shape HCC patient prognosis.

**Table 4:** Correlation analysis between NCAPH and biomarkers of immune cells in hepatic cell cancer.

**Immune Cell**	**Gene**	**Correlation Coef.**	***p***
B cell	**CD19**	**0.228475**	**8.09E-06**
	CD79A	0.086287	0.095665
CD8+ T cell	CD8A	0.156068	0.002497
	CD8B	0.136268	0.00832
CD4+ T cell	CD4	0.158395	0.002146
M1 macrophage	NOS2	−0.04473	0.388393
	**IRF5**	**0.325369**	**1.46E-10**
	PTGS2	0.019707	0.704033
M2 macrophage	CD163	0.063616	0.219562
	VSIG4	0.075547	0.144727
	MS4A4A	0.069518	0.179662
Neutrophil	CEACAM8	0.160825	0.001808
	**ITGAM**	**0.293687**	**8.36E-09**
	CCR7	0.004526	0.930457
Dendritic cell	HLA-DPB1	0.120851	0.019442
	HLA-DQB1	0.123065	0.017312
	HLA-DRA	0.154326	0.002793
	HLA-DPA1	0.118242	0.02224
	CD1C	0.027322	0.598393
	**NRP1**	**0.217181**	**2.37E-05**
	**ITGAX**	**0.272233**	**9.99E-08**

### MELK and HCC immune checkpoints

**Figure 9:**
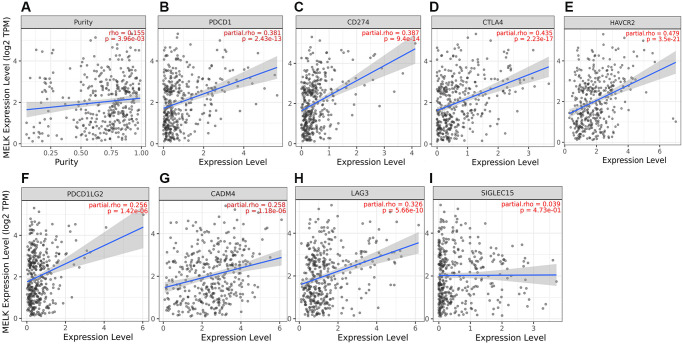
**MELK levels and immune checkpoint expression.** (**A**) Correlation of MELK with PD-1, adjusted for purity by TIMER, (**B**) Correlation with PDL1, (**C**) Correlation with CTLA-4, (**D**) Correlation with HAVCR2, (**E**) Correlation with PDCD1, (**F**) Correlation with PDCD1LG2, (**G**) Correlation with TIGIT, (**H**), Correlation with LAG3, (**I**) Correlation with SIGLEC15.

The potential links between MELK and the immune checkpoints PD1/PD-L1 (CD274), CTLA-4, HAVCR2, PDCD1, PDCD1LG2, TIGIT (CADM4), LAG3, and SIGLEC15 were assessed using TIMER. After adjustment for tumor purity, a significant positive correlation was observed between MELK and the expression of these immune checkpoint genes (Figure 9A–9I). This indicated a positive relationship between MELK levels and all the immune checkpoints, implicating MELK in HCC immune escape.

## DISCUSSION

HCC is notable for its high mortality. It is hoped that clarification of its etiology may assist in the development of treatment targets as well as identifying biomarkers to assist diagnosis and prognosis. The involvement of MELK in many human cancers, including HCC, has been documented [18]. However, our understanding of its role in HCC remains largely undetermined. Here, we first analyzed MELK levels in a variety of cancers using data from the TCGA, followed by verification using GEPIA. Further evaluation demonstrated a link between raised MELK levels and poor outcomes in HCC. We also found that the noncoding RNAs LINC00265/has-miR-101-3p mediated upregulation of MELK.

Xia et al. proposed that MELK promotes endometrial carcinoma progression through the E2F1/MELK/ mTORC1/2 axis [19]. Overexpression of MELK was found to correlate with early tumor recurrence and poor patient survival in HCC. The overexpression of MELK in HCC samples strongly correlated with the expression of cell cycle- and mitosis-related genes, while silencing MELK inhibited the cell growth, invasion, stemness, and tumorigenicity of HCC cells by inducing apoptosis and mitosis [19]. These findings, together with our own, suggest that MELK functions as an oncogene in HCC. Furthermore, the crosstalk between ncRNAs in modulating gene expression through the ceRNA process has been well documented [20, 21].

We used seven algorithms, namely, PITA, RNA22, miRmap, microT, miRanda, PicTar, and TargetScan, to identify potential MELK-binding miRNAs. The miRNA has-miR-101-3p was found to be the most likely candidate and may thus be a likely HCC biomarker. miR-101-3p target molecules have been implicated in HCC carcinogenesis [22]. Further investigation indicated that miR-101-3p has tumor-suppressing functions upstream of MELK. This miRNA has been previously found to block HCC proliferation and migration. The ceRNA hypothesis [23] suggests that lncRNAs interacting with the miR-101-3p/MELK axis may have oncogenic functions in HCC. We next identified 25 lncRNAs likely to function upstream of the miR-101-3p/MELK axis, with further examination revealing MIR3142HG and LINC00265 to be the most probable. LINC00265 has been linked to the progression of various cancers, including HCC [24, 25]. Taken together, MIR3142HG and the LINC00265/has-miR-101-3p/MELK axis were determined to be potential modulatory factors in HCC. The involvement of this axis explains both MELK’s elevated expression and links to poor survival outcomes in HCC, together with the reduction in miR-101-3p.

We did not observe any relationship between MELK levels and copy number variations in HCC. The role of DNA methylation in gene expression is well documented [26, 27] and it is possible that this accounts for the dysfunctional expression of MELK in HCC. Hypomethylation of MELK was seen in HCC tissue, which supports the observation of elevated MELK levels together with elevated levels of the DNA methyltransferases (DNMT1, DNMT3A, and DMNT3B). Certain methylation sites were also observed to correlate with HCC patient outcomes.

Immune cell infiltration is known to influence the effectiveness of cancer treatments, including immunotherapy and adjuvant therapy, as well as patient outcomes [28, 29]. Here, we found a positive relationship between MELK and TILs, including B cells, CD8+ T cells, CD4+ T cells, macrophages, neutrophils, and dendritic cells, in HCC. In addition, there was an association between MELK and biomarkers of these cells. MELK was associated with poorer HCC patient survival and neutrophil and macrophage levels. These observations suggest that immune infiltration may be at least partially responsible for MELK’s role in HCC carcinogenesis.

Successful immunotherapy relies not only on adequate numbers of TILs but also on the level of immune checkpoint expression [30, 31]. For this reason, we examined the link between MELK and immune checkpoints, observing that raised levels of MELK were linked to all the examined checkpoints, implying that targeting MELK could enhance the effectiveness of immunotherapy.

Here, we identified a ceRNA-based MIR3142HG and LINC00265/has-miR-101-3p/MELK axis that may be a potential prognostic biomarker in clinical applications. Nevertheless, several limitations must also be noted. First, the binding affinities of the lncRNAs, miRNAs, and mRNAs obtained from the database require further experimental investigation. Second, further experimental verification of the function and mechanism of the MIR3142HG and LINC00265/has-miR-101-3p/MELK axis in HCC is required.

In conclusion, we demonstrated elevated expression of MELK in various human cancers, including HCC, which was linked to poor survival outcomes. We further showed that MIR3142HG and the LINC00265/ has-miR-101-3p axis acted as upstream regulators of MELK. MELK may thus be a significant prognostic indicator of HCC outcome. Nevertheless, these findings require verification by further research and future clinical trials.
